# Does a pretreatment with a dentine hypersensitivity mouth-rinse compensate the pain caused by professional mechanical plaque removal? A single-blind randomized controlled clinical trial

**DOI:** 10.1007/s00784-020-03643-4

**Published:** 2020-10-23

**Authors:** Sonja H. M. Derman, Eva-Maria Lantwin, Anna Greta Barbe, Michael J. Noack

**Affiliations:** Department of Operative Dentistry and Periodontology, University of Cologne, Kerpener Str. 32, 50931 Cologne, Germany

**Keywords:** Professional mechanical plaque removal, SRP, Dipotassium oxalate, Arginine, Mouth-rinse, Pain, Periodontitis

## Abstract

**Objectives:**

Pain is affecting acceptance of supportive periodontal therapy and primary periodontitis prevention. Our objective was to evaluate the efficacy of a 1-week pre-treatment use of dentinal-hypersensitivity-reducing mouth-rinses (DHM) in periodontal maintenance (SPT) or dental prophylaxis patients.

**Material and methods:**

One hundred fifty-five participants attending for professional mechanical plaque removal (PMPR) were randomly assigned to use a mouth-rinse twice daily for 1 week prior to their next PMPR. Rinses were containing either potassium oxalate (*n* = 52), arginine (*n* = 52), or herbal extracts (*n* = 51). At baseline and reassessment, procedural pain was assessed by visual analogue scale (VAS) and verbal rating scale (VRS). Self-reported efficacy was documented.

**Results:**

No inter-group differences were estimated between both test groups and the control for baseline and reassessment means (VAS, VRS). In the SPT group, VAS reduction and self-reported efficacy were found (*p* < 0.05).

**Conclusion:**

The 1-week use of DHM failed to show a predictable effect on discomfort during PMPR overall. Around 20% of the patients showed a quantifiable benefit from both test mouth-rinses, whereas more than 50% reported a subjective pain reduction. Focusing patients undergoing supportive periodontal therapy, quantifiable effects were found for both test groups. From a patient’s point of view, DHM might be a suitable adjunct to enhance procedural comfort, especially in patients with a history of periodontitis.

**Clinical relevance:**

The 1-week use of the dentinal-hypersensitivity-reducing mouth-rinses prior to professional-mechanical-plaque-removal showed to be a suitable adjunct to enhance procedural comfort during instrumentation, especially in patients undergoing supportive periodontal therapy.

**Registration number:** DRKS00010811

**Electronic supplementary material:**

The online version of this article (10.1007/s00784-020-03643-4) contains supplementary material, which is available to authorized users.

## Introduction

The medical benefits of professional mechanical plaque removal (PMPR) are well documented for primary and secondary prevention of periodontitis [1–4]. Focusing on the patient’s comfort, the main objective should be the reduction of procedural pain. PMPR, as performed for periodontal supportive therapy or dental prophylaxis, causes pain or discomfort for most patients [5, 6]. This may lead to non-attendance for recall appointments [7, 8]. In periodontal patients, this avoidance induces periodontal reinfection and may contribute—at worst—to tooth loss [9]. Additionally, the grade of inflammation correlates with the pain level during probing and PMPR [10–12]. Lastly, more inflammation leads to a more painful PMPR.

A source of discomfort or pain during PMPR is the mechanical irritation of the dentin-pulp complex. The pain origin is similar to dentine hypersensitivity—the hydrodynamical theory [13]. In the case of a thermal, a tactile, or a chemical irritation, a fluid flow in the dentine tubules occurs and pulpal pain receptors are stimulated. While more and wider opened dentine tubules are found in hypersensitive teeth, the same is found after periodontal therapy [14]. Our hypothesis was that dentine-hypersensitivity mouth-rinses offer a simple solution to enable patients to reduce procedural pain or discomfort during PMPR. As we hypothesized that a history of periodontitis and self-reported pain sensitivity may have an impact on pain perception, we used this parameter for block-randomization and subgroup analysis.

Therefore, the aim of this study was to evaluate the efficacy on procedural pain during PMPR of a 1-week use of two mouth-rinses designed to relieve dentin hypersensitivity prior to PMPR on pain or discomfort in patients ongoing primary or secondary periodontal prevention. Primary outcome was procedural pain during PMPR measured by VAS and VRS, and secondary outcomes were self-reported efficacy and safety.

## Materials and method

### Study population and methodology

This was a randomized, clinical, single-blind, controlled, parallel-group, investigator-initiated trial conducted in Germany to evaluate the impact of a 1-week pre-operative use of two mouth-rinses designed for dentine hypersensitivity (test 1: DPOX, Listerine sensitive professional, Johnson and Johnson Consumer & Personal products Worldwide, Skillmann, NJ, USA, test 2: ARGI, Elmex sensitive professional, Colgate Palmolive, New York City, NY, USA, control: CRTL, Nanaminze mouth-rinse, Alverde Naturkosmetik, Karlsruhe, D) on pain or discomfort caused by PMPR in maintenance patients (risk-based periodontal supportive therapy or dental prophylaxis). All examinations were carried out at the Department of Operative Dentistry and Periodontology at the University of Cologne. The study was approved by the local ethics review board of the University of Cologne (no. 16-257) and registered (DRKS00010811). All participants gave written informed consent before study-related procedures were carried out.

Participants were individually instructed in the use of the mouth-rinses, and written instructions were also provided. The participants were instructed not to use any other kind of mouth-rinse and continue the use of their pre-study dentifrice, toothbrush, and interdental cleaning routine.

### Sample size calculation

A study of a continuous response variable from independent control and experimental subjects with one control per experimental subject was planned. In a pre-study, the response within each subject group was normally distributed with standard deviation 1. If the true difference in the experimental and control means is 0.5, 64 experimental and 64 controls are needed to reject the null hypothesis that the population means of the experimental and control groups are equal with the power 0.8. The type I error probability associated with this test of this null hypothesis is 0.05. A priori, an interim analysis was planned after 12 months duration of recruitment and at least 50% of participants included.

### Selection of participants

After the ethical and organizational tasks were fulfilled, the screening began in September 2016. Participants were recruited in the Department of Operative Dentistry and Periodontology at the University Hospital Cologne. Inclusion criteria were (1) risk-based periodontal maintenance or dental prophylaxis patient (patients attending for primary or secondary periodontitis prevention, initial diagnosis either gingivitis or periodontitis), (2) recall frequency twice a year (or more), (3) recall appointment third or more, and (4) adult patients (> 18 years old). Exclusion criteria were (1) presence of acute dental pain, pulpitis, or other acute dental infections; (2) caries lesions with invasive treatment need; (3) antibiotic therapy (up to 7 days prior to the study appointments); (4) history of allergic or undesirable reactions to the test products or ingredients; (5) long-term medication with analgetics, (6) diseases or medication influencing inflammation or the immune system (including anti-inflammatory drugs); (7) abuse to alcohol; and (8) pregnant or lactating women.

### Randomization and allocation concealment

Block randomization (strata: self-reported pain sensitivity and diagnosis) into three groups was provided by the Institute for Medical Statistics, Informatics and Epidemiology, University of Cologne (https://prod.tenalea.net/zkskoeln/dm/). Allocation concealment was achieved by having a person not involved in clinical examination (L. V.) distributing the mouth-rinses and giving instructions for use. Examiners were blinded regarding the mouth-rinse used.

### Baseline appointment—PMPR 1

First, papilla bleeding index (PBI) and plaque index (PI) were obtained as described in detail elsewhere [15, 16]. PMPR was performed using an ultrasonic scaler (Cavitron with Slimline inserts, Dentsply Sirona, Charlotte, NC, USA) to remove coarse calculus and disrupt the biofilm followed by Gracey curettes (Hu-Friedy, Chicago, IL, USA) and polishing (NUPRO Prophy Paste, Dentsply Sirona, Charlotte, NC, USA) at all accessible supra- and subgingival tooth and root surfaces. Patients were individually instructed by the use of a 0 to 100 visual analogue scale (VAS) and a five-step verbal rating scale (VRS) to record the experienced pain levels. Additionally, the modified dental anxiety scale (MDAS) was assessed by baseline questionnaire [17].

### Rinsing pretreatment

Participants started to use the assigned mouth-rinse 1 week prior to the second PMPR appointment twice daily—in the morning and the evening—for 1 min directly after toothbrushing. The amount of mouth-rinse per use followed the application instructions of the manufacturers (DPOX 20 ml, ARGI 10 ml, CRTL 10 ml). Participants were instructed not to use any other kind of mouth-rinse and continue the use of their pre-study dentifrice, toothbrush, and interdental cleaning routine. They were asked to complete a rinsing diary and document each use of the mouth-rinse including time of use and amount. The diaries were collected and checked for completeness after second study appointment to ensure adherence to the study protocol.

### Study appointment—PMPR 2

Indices and PMPR were proceeded as prescribed above. Additionally, intra-oral soft tissue examination was performed (safety protocol). Again, all participants filled in a questionnaire regarding their self-reported efficacy of the assigned mouth-rinse and the procedural pain via VAS and VRS.

### Statistical analysis

Between groups differences (VAS, VRS) were evaluated for baseline, study appointment, and the treatment effect by one-way ANOVA. Within group analysis of the treatment effect was performed by Wilcoxon signed-rank test. Pearson’s chi-square test was used for dichotomous parameters. Additionally, subgroup analyses dividing the groups by initial diagnosis were performed as described above. All analyses were carried out at participant level (unit of analysis) using SPSS Statistics 24 Software (SPSS Inc., Chicago, IL, USA). *p* values ≤ 0.05 were considered to indicate statistical significance. Data were typed twice to minimize data entry mistakes. Missing data (drop out) were handled by the last-observation-carried-forward method (LOCF).

## Results

**Fig. 1:**
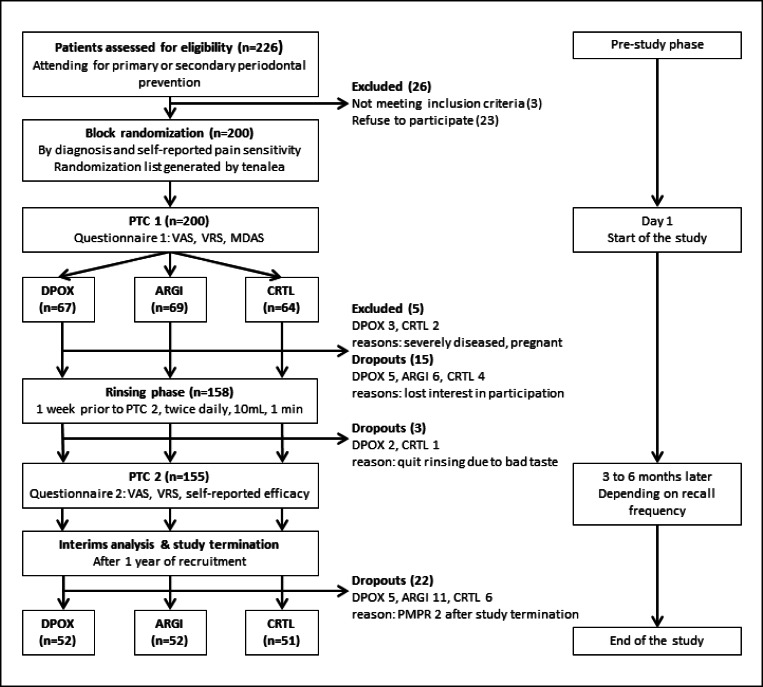
Study flow chart (VAS, visual analogue scale; VRS, verbal rating scale; MDAS, modified dental anxiety scale; DPOX, dipotassium oxalate = test 1; ARGI, arginine = test 2; CRTL, control; PMPR, professional mechanical plaque removal)

A total of 155 participants (81 females, 74 males) completed the trial (Table 1, Fig. 1). The interim analysis after 12 months study duration showed that there was no statistical significance regarding the differences between products. Statistical evaluation pointed out that also with inclusion of further patients, there was no possibility to reach statistical significance. Therefore, the study was terminated. Data presented are obtained by per protocol (PP) analysis. Intention to treat analysis was performed confirming robustness of the results obtained by PP analysis. Patients’ characteristics were distributed equally between the groups, except for age (Table 1).

**Table 1:** Distribution of patients’ characteristics

	DPOX Test 1 *n* = 52	ARGI Test 2 *n* = 52	CRTL Control *n* = 51	*p* ANOVA
	*n* Mean ± SD Min–max			
Age	52 58.4 ± 13.7 23–81	52 52.7 ± 15.2 19–85	51 61.0 ± 13.0 35–86	*0.010*
mDAS	52 8.3 ± 3.1 5–19	51 8.8 ± 3.4 5–17	51 8.6 ± 3.7 5–21	0.704
PI	52 0.2 ± 0.3 0.0–1.4	52 0.2 ± 0.4 0.0–1.4	51 0.1 ± 0.2 0.0–1.1	0.263
PBI	52 0.3 ± 0.2 0.0–1.3	52 0.3 ± 0.3 0.0–1.1	51 0.3 ± 0.4 0.0–2.3	0.424
BOP	26 3.3 ± 5.0 0.0–16.7	23 4.0 ± 6.0 0.0–24.5	22 1.1 ± 1.8 0.0–6.7	0.093
	*n* (%)			Pearson’s chi-square
Non-smoker Smoker	47 (90) 5 (10)	49 (94) 3 (6)	49 (96) 2 (4)	0.486
Male Female	22 (42) 30 (58)	27 (52) 25 (48)	25 (49) 26 (51)	0.603
Gingivitis Periodontitis	21 (40) 31 (60)	21 (40) 31 (60)	18 (35) 33 (65)	0.830
Pain sensitivity self-reported				
No Yes	11 (21) 41 (79)	9 (17) 43 (83)	7 (14) 44 (86)	0.610
Pain sensitivity—estimated by dental hygienist				
No Moderate Severe	13 (25) 37 (71) 2 (4)	16 (31) 31 (61) 4 (8)	21 (41) 28 (55) 2 (4)	0.373

*mDAS*, modified dental anxiety scale; *PI*, plaque index; *PBI*, papilla bleeding index; *BOP*, bleeding on probing

*p* values in italic indicate statistically significant differences between the groups

### VAS

No inter-group differences could be estimated between test-groups and placebo (ANOVA *p* > 0.05, Table 2). Regarding the intra-group treatment effect, dipotassium oxalate proved a pain reduction ability (Wilcoxon signed-rank test *p* = 0.000) in contrast to arginine and control (*p* > 0.05). Pointing out the responder analysis, dipotassium oxalate showed the highest amount of responders and the lowest amount of non-responders compared to arginine and control group (Pearson’s chi-square test *p* = 0.013, Table 2). Subgrouping by diagnosis revealed a superiority in the responder-analysis for DPOX (chi-square test *p* = 0.020), accompanied by the largest VAS reduction (Delta VAS, ANOVA *p* = 0.049) and a superior intra-group treatment effect (Wilcoxon signed-rank test *p* = 0.001) in periodontitis patients. None of these effects was found in the gingivitis group (Table 5).

**Table 2:** Inter- and intragroup changes in pain perception by VAS and VRS during PMPR

	DPOX Test 1 *n* = 52	ARGI Test 2 *n* = 52	CRTL Control *n* = 51	*p* ANOVA
	Mean ± SD Min–max			
Visual analogue scale (VAS)				
VAS pre	31.6 ± 23.4 0 to 83	28.9 ± 22.2 0 to 84	28.9 ± 24.6 0 to 94	0.793
VAS post	22.6 ± 17.3 0 to 60	26.4 ± 20.4 0 to 78	27.8 ± 23.3 0 to 73	0.414
Wilcoxon signed-rank test	*0.000*	0.283	0.454	
Responder	30 (58%)	25 (48%)	26 (51%)	
Equal	19 (36%)	11 (21%)	10 (20%)	
Non-responder	3 (6%)	16 (31%)	15 (29%)	
Pearson’s chi-square	*0.010*			
Delta VAS	9.0 ± 17.9 − 50 to 65	3.2 ± 25.0 − 68 to 46	1.8 ± 24.3 − 48 to 63	0.233
Verbal rating scale (VRS)				
VRS pre	1.4 ± 0.9 0 to 4	1.5 ± 0.8 0 to 3	1.4 ± 0.9 0 to 3	0.855
VRS post	1.2 ± 0.7 0 to 3	1.3 ± 0.7 0 to 3	1.4 ± 0.8 0 to 3	0.679
Wilcoxon signed-rank test	0.197	0.058	0.797	
Responder	22 (42%)	23 (44%)	27 (53%)	
Equal	28 (54%)	28 (54%)	21 (41%)	
Non-responder	2 (4%)	1 (2%)	3 (6%)	
Pearson’s chi-square	0.582			

*p* values in italic indicate statistically significant differences between the groups

Responder: delta VAS ≥ 5 or delta VRS ≥ 1

Equal: delta VAS 4 to − 4 or delta VRS = 0

Non-responder: delta VAS ≤ − 5 or delta VRS ≤ 1

### VRS

No inter-group and intra-group differences could be estimated between test groups and placebo (ANOVA *p* = 0.679, Wilcoxon signed-rank test *p* > 0.05, Table 2). No differences were found in the VRS responder analysis between groups (Pearson’s chi-square test *p* = 0.582). Subgrouping by diagnosis revealed the same results for gingivitis and periodontitis patients (Table 5). VRS scores reporting “no” or only “mild pain” increased in both test groups by 21%, whereas the change in the control group was merely 4% (Table 3).

**Table 3:** Distribution of pain perception during PMPR by verbal rating scale (VRS)

Pain (VRS)	DPOX *n* = 52		ARGI *n* = 52		CRTL *n* = 51	
	Pre *n* (%)	Post *n* (%)	Pre *n* (%)	Post *n* (%)	Pre *n* (%)	Post *n* (%)
No	9 (17)	7 (13)	6 (12)	5 (10)	9 (18)	7 (14)
Mild	19 (37)	27 (52)	20 (38)	32 (61)	20 (39)	24 (47)
Moderate	20 (38)	17 (33)	22 (42)	12 (23)	16 (31)	15 (29)
Severe	3 (6)	1 (2)	4 (8)	3 (6)	6 (12)	5 (10)
Worst	1 (2)	0 (0)	0 (0)	0 (0)	0 (0)	0 (0)
Percentage %						
No to mild pain	54	65	50	71	57	61
Moderate to severe	46	35	50	29	43	39
Delta	11		21		4	

### Self-reported efficacy

More than 50% of the participants mentioned self-reported efficacy on pain reduction during PMPR in both test mouth-rinses (*p* = 0.026, Table 4). Again, dipotassium oxalate pointed out with the highest amount of self-reported efficacy and the lowest amount of non-responders. For patients ongoing periodontal supportive therapy, both test groups showed higher amount of responders compared to the control group (*p* = 0.025). In gingivitis patients, no differences were shown (Table 5).

**Table 4:** Self-reported efficacy (n and %), overall and subgrouping by initial diagnosis

	Yes *n* (%)	No *n* (%)	Indecisive *n* (%)	*p* value*
Overall	70 (45)	35 (23)	49 (32)	
DPOX	30 (58)	8 (15)	14 (27)	*0.026*
ARGI	26 (51)	10 (20)	15 (29)	
CRTL	17 (33)	14 (28)	20 (39)	
Periodontitis	43 (46)	19 (20)	32 (34)	
DPOX	19 (61)	3 (10)	9 (29)	*0.025*
ARGI	16 (53)	5 (17)	9 (30)	
CRTL	8 (24)	11 (33)	14 (43)	
Gingivitis	27 (45)	16 (27)	17 (28)	
DPOX	11 (52)	5 (24)	5 (24)	0.814
ARGI	10 (48)	5 (24)	6 (28)	
CRTL	6 (33)	6 (33)	6 (33)	

*Pearson’s chi-square

*p* values in italic indicate statistically significant differences between the groups

**Table 5:** Diagnosis related distribution of inter- and intragroup changes of pain perception (via VAS and VRS)

Gingivitis	DPOX Test 1 *n* = 21	ARGI Test 2 *n* = 21	CRTL Control *n* = 18	*p* ANOVA	Periodontitis	DPOX Test 1 *n* = 31	ARGI Test 2 *n* = 31	CRTL Control *n* = 33	*p* ANOVA
	Mean ± SD Min–max					Mean ± SD Min–max			
Visual analogue scale									
VAS pre	25.5 ± 22.6 0 to 83	27.8 ± 20.3 0 to 70	26.8 ± 20.6 5 to 65	0.943	VAS pre	35.7 ± 23.3 0 to 73	29.6 ± 23.6 0 to 84	30.0 ± 26.8 0 to 94	0.556
VAS post	17.5 ± 16.3 0 to 60	23.7 ± 19.5 0 to 78	19.6 ± 22.8 0 to 69	0.582	VAS post	26.1 ± 17.4 0 to 56	28.2 ± 21.1 0 to 73	32.5 ± 22.8 0 to 73	0.582
Wilcoxon signed-rank test	0.153	0.513	0.085		Wilcoxon signed-rank test	*0.001*	0.271	0.829	
Responder	9 (43%)	8 (38%)	10 (56%)		Responder	18 (58%)	18 (58%)	13 (39%)	
Equal	10 (48%)	6 (29%)	5 (28%)		Equal	12 (39%)	5 (16%)	9 (27%)	
Non-responder	2 (9%)	7 (33%)	3 (17%)		Non-responder	1 (3%)	8 (26%)	11 (33%)	
Pearson’s chi-square	0.260				*0.020*				
Delta VAS	8.0 ± 19.9 − 30 to 65	4.1 ± 21.7 − 30 to 46	7.2 ± 25.1 − 47 to 63	0.328	Delta VAS	9.6 ± 16.7 − 50 to 37	2.6 ± 27.4 − 68 to 38	− 1.2 ± 23.8 − 48 to 44	0.*049*
Verbal rating scale									
VRS pre	1.1 ± 0.7 0 to 3	1.4 ± 0.9 0 to 3	1.2 ± 0.8 0 to 3	0.509	VRS pre	1.6 ± 1.0 0 to 4	1.5 ± 0.8 0 to 3	1.5 ± 1.0 0 to 3	0.860
VRS post	0.9 ± 0.6 0 to 2	1.2 ± 0.6 0 to 3	1.2 ± 0.9 0 to 3	0.375	VRS post	1.5 ± 0.7 0 to 3	1.3 ± 0.8 0 to 3	1.5 ± 0.8 0 to 3	0.616
Wilcoxon signed-rank test	0.305	0.248	0.873		Wilcoxon signed-rank test	0.400	0.132	0.835	
Responder	7 (33%)	9 (43%)	8 (44.5%)		Responder	15 (48.5%)	14 (45%)	19 (58%)	
Equal	13 (62%)	12 (57%)	8 (44.5%)		Equal	15 (48.5%)	16 (52%)	13 (39%)	
Non-responder	1 (5%)	0 (0%)	2 (11%)		Non-responder	1 (3%)	1 (3%)	1 (3%)	
Pearson’s chi-square	0.505				Pearson chi-square	0.896			

*p* values in italic indicate statistically significant differences between the groups

### Safety

No adverse events by means of soft tissue irritation or allergic reactions were clinically found or reported by the participants. Three participants (DPOX 2, CRTL 1) quit using the assigned mouth-rinse because of bad taste.

## Discussion

To our knowledge, this randomized controlled trial is the first to evaluate the efficacy of a 1-week use of mouth-rinses designated for dentinal hypersensitivity as pretreatment to control pain and discomfort during professional mechanical plaque control in patients undergoing primary or secondary periodontal prophylaxis. Even if the dentinal-hypersensitivity-reducing mouth-rinses were not able to achieve a predictable impact on discomfort during PMPR for all patients, dipotassium oxalate proved a pain reduction ability overall and in the periodontitis group but not for gingivitis patients.

Focusing VAS responder-analysis, all groups showed around 50% responders, but the dipotassium oxalate users showed noticeable fewer non-responders with 6% than the others (ARGI 29%, CRTL 28%). In patients undergoing periodontal supportive therapy, both test groups showed a higher amount of VAS responders which was not found in gingivitis patients.

Also, both test groups showed a higher amount of self-reported efficacy, and again the dipotassium group pointed out with the best results. Despite the fact that a predictable impact on pain experience measured by VAS was not found, the subjective efficacy was reported by more than 50% of the participants in the test groups. Thus, our hypothesis was not supported by the primary outcome but proved for self-reported efficacy. Again, this effect was quantifiable for periodontitis patients but limited in the gingivitis group. Even if the measurable pain reduction could not be found, this patient-reported outcome (PRO) is a very valuable information as it reflects the individual perception of the participants. The recent EFP position paper focusing on endpoints of active periodontal therapy demands PROs to be included in studies additional to clinical measurements [18]. Patients need tangible and perceptible outcomes to accept and adhere to the lifelong prevention or treatment programs. And procedural pain is found to be a common reason to avoid appointments for periodontal supportive care not only in anxious patients [6, 19].

The overall analysis and subgrouping by diagnosis revealed a higher efficacy of the mouth-rinse pretreatment in the periodontitis-group, especially for DPOX. As expected, the occlusion-based active ingredients proved a higher impact on pain reduction in periodontal patients. This may be explained by the already experienced attachment loss accompanied by a higher amount of open dentine tubules. No quantifiable effect could be found for gingivitis patients. For them, procedural pain during PMPR may rather be soft tissue-associated or caused by pain peak sites with dentine hypersensitivity. The prevalence of dentine hypersensitivity is high and affects around one-third of the patients, even in young adults [20, 21]. One possible explanation for the lower efficacy of the rinsing pretreatment is that the open dentine tubules in sensitive teeth are harder to occlude due to habits (parafunction) and lifestyle (acidic diet, beverages) favoring the development of dentine hypersensitivity and thus reducing the occlusion-based effects [21].

During PMPR both—hard and soft tissues—are stimulated and therefore both may be a source of experienced discomfort with individual distribution. As intrapocket anesthesia is a well-documented option to overcome soft tissue pain in a non-invasive manner, hard tissue–related sensations are predominantly controlled by invasive injection anesthesia or in-office use of medical products [22–25].

A meta-analysis focusing the use of desensitizing mouth-rinses to control dentin hypersensitivity was performed in 2017 [26]. Due to the inclusion criteria, none of the trials reporting results of ingredients used in our trial was included. Nevertheless, both test group ingredients’ efficacy to reduce dentine hypersensitivity was proven in multiple studies [27–32].

We have also carried out a gender-specific subgroup analysis (supplement 2). The outcomes support the overall results, even if the female participants tend to have higher values in VAS pre (significant for DPOX and overall, but possibly impacted by the uneven distribution). Neither the VAS post nor the Delta VAS show any differences overall and in the groups between the sexes.

To our knowledge, this study is the first to evaluate the impact of products accessible and used by patients to influence the pain and discomfort during PMPR. Regarding patient-centered outcomes and taking patient autonomy into account are key points for long-term patient relationships. Our idea of an indication-expansion of mouth-rinses designated for dentinal hypersensitivity as pretreatment prior to PMPR to reduce procedural discomfort is one step forward to strengthen the self-responsibility of patients.

### Limitations

In this single-blind design, participants were aware of products used. Dropouts were evenly distributed between the test products and control even if two expensive pharmacy or rather over-the-counter products were compared with an inexpensive vegan mouth-rinse (own brand of German drugstore). Both test products belong to well-known brands from the healthcare sector. This may have triggered a positive expectation in patients, which may have been reflected in an overly good evaluation of self-reported efficacy.

Based on the sample size calculation, we needed 64 participants per group and included 10% more for drop-outs. During the study, every fifth participant dropped out. That may be attributed to long period between the PMPR appointments with 3 to 6 months depending on risk-based recall frequency (see Fig. 1). Having more drop-outs occurred than expected is the explanation for the uneven distribution of groups after termination of the study.

A possible interaction of the dentifrice with the mouth-rinses could not be excluded. Patients were asked to continue the use of their preferred dentifrices during the study. Therefore, the impact of dentifrices is distributed intra-individual equally to both appointments which minimizes the possible bias.

One-third of the controls exhibited a self-reported efficacy on reduction of procedural pain during PMPR after rinsing pretreatment. That may be attributed to either the placebo or the *Hawthorne* effect [33, 34]. Both are unintended consequences of research participation. These results may although have been found in both test groups which may have weakened the subjective efficacy. None of the ingredients of the control mouth-rinse was discussed in literature to have a possible desensitizing effect. The *Hawthorne* effect was successfully used intentionally to improve oral home care in orthodontic patients [34]. As two treatment appointments (with and without mouth-rinse pretreatment) were evaluated in our study, this possible impact could be excluded.

It is well-known that the grade of inflammation correlates with the pain during instrumentation [10, 12]. We eliminated this source of bias by selecting participants experienced in PMPR attending for primary or secondary prevention reflected by low levels of marginal inflammation (PBI) and bleeding on probing (BOP). A recent meta-analysis showed that aging decreases the sensitivity for pain of low intensity, especially apparent for heat pain and pain of the head [35]. It is not possible to quantify whether and to what extent this may have an impact on our results. The proportion of smokers in our population was very low (6%). Even if consistently observed in animal research, demonstration of nicotine’s antinociceptive effect in humans has proven elusive [36]. Smoking and nicotine use have a bidirectional link to chronic pain and depression [37]. Due to the low number of smokers and the current scientific data, we have not carried out this subgroup analysis.

Pain experience is influenced not only by mechanical stimuli but also by psychological factors, patients’ pain history, and former experiences. Self-reported pain sensitivity and dental anxiety were additionally recorded, and no differences were estimated for baseline means. Thus, an unequal distribution of these factors as potential source of bias could be excluded. An influence of periodontal diagnosis can also be ruled out, as these are evenly distributed between the groups (*p* = 0.542, supplement 1).

As the subgroup analysis by initial diagnosis revealed a higher efficacy of this pretreatment regarding pain reduction in patients with a history of periodontitis and the sample size calculation was accomplished only for the whole study population, the strong impact of this factor should be approved by further studies with narrowly defined inclusion criteria.

## Conclusions

The 1-week use of dentinal-hypersensitivity-reducing mouth-rinses failed to show a predictable impact on discomfort during PMPR for all patients. Overall, around 20% of the patients showed a quantifiable benefit from both test mouth-rinses, whereas more than 50% reported a subjective pain reduction. But for patients undergoing supportive periodontal therapy, quantifiable effects were found for dipotassium oxalate and arginine containing mouth-rinses for both objective and self-reported efficacy. From a patient’s point of view, the 1-week use of the dentinal-hypersensitivity-reducing mouth-rinses before PMPR might be a suitable adjunct to enhance procedural comfort especially in periodontal patients. Overall and for gingivitis patients, the effect depends on one individuals’ response to the product used. In addition to research objectives such as optimizing the treatment outcome, there is a tremendous need for research focusing the improvement of patients’ comfort during regular professional mechanical plaque removal. From a patients’ point of view, a sufficient pain/discomfort management does not only mean a better service quality for each single appointment but is also an important point for improving the willingness to adhere to regular and life-long primary or secondary prevention of periodontitis.

## Electronic supplementary material

ESM 1 (DOCX 14 kb)

ESM 2 (DOCX 15 kb)

ESM 3 (DOCX 11 kb)
