# Supplementary material for: Does a pretreatment with a dentine hypersensitivity mouth-rinse compensate the pain caused by professional mechanical plaque removal? A single-blind randomized controlled clinical trial
Source: Clin Oral Investig. 2020 Oct 23;25(5):3151–60. doi: 10.1007/s00784-020-03643-4 (PMC8060178; doi:10.1007/s00784-020-03643-4)
Supplement: Supplementary file 1 — (DOCX 14 kb) [file 784_2020_3643_MOESM1_ESM.docx]

**Table S1**: Distribution of periodontal diagnosis by groups

| **Localization** | **Stage** | **Grade** | **Overall**  N=94  n (%) | **DPOX**  N=31  n (%) | **ARGI**  N=30  n (%) | **CRTL**  N=33  n (%) | *p** |
| --- | --- | --- | --- | --- | --- | --- | --- |
| generalized | I | A | 3 (3) | 1 (3) | 1 (3) | 1 (3) | .542 |
|  |  | B | - | - | - | - |  |
|  |  | C | - | - | - | - |  |
|  | II | A | 8 (9) | 3 (10) | 4 (13) | 1 (3) |  |
|  |  | B | 23 (24) | 8 (26) | 8 (27) | 7 (21) |  |
|  |  | C | - | - | - | - |  |
|  | III | A | - | - | - | - |  |
|  |  | B | 26 (28) | 6 (19) | 5 (17) | 15 (46) |  |
|  |  | C | 12 (23) | 4 (13) | 3 (10) | 5 (15) |  |
|  | IV | A | - | - | - | - |  |
|  |  | B | 1 (1) | 1 (3) | - | - |  |
|  |  | C | 13 (14) | 5 (16) | 5 (17) | 3 (9) |  |
| localized | I | A | - | - | - | - |  |
|  |  | B | - | - | - | - |  |
|  |  | C | - | - | - | - |  |
|  | II | A | 1 (1) | - | 1 (3) | - |  |
|  |  | B | 4 (4) | 2 (7) | 2 (7) | - |  |
|  |  | C | - | - | - | - |  |
|  | III | A | - | - | - | - |  |
|  |  | B | 2 (2) | 1 (3) | - | 1 (3) |  |
|  |  | C | - | - | - | - |  |
|  | IV | A | - | - | - | - |  |
|  |  | B | - | - | - | - |  |
|  |  | C | 1 (1) | - | 1 (3) | - |  |

* Pearson Chi Square Test
