# Supplementary material for: Does a pretreatment with a dentine hypersensitivity mouth-rinse compensate the pain caused by professional mechanical plaque removal? A single-blind randomized controlled clinical trial
Source: Clin Oral Investig. 2020 Oct 23;25(5):3151–60. doi: 10.1007/s00784-020-03643-4 (PMC8060178; doi:10.1007/s00784-020-03643-4)
Supplement: Supplementary file 2 — (DOCX 15 kb) [file 784_2020_3643_MOESM2_ESM.docx]

**Table S2**: inter- and intragroup changes in pain perception by VAS and VRS during PMPR with subgrouping by gender

|  | **VAS pre**  Mean ± SD  (min – max) | **VAS post**  Mean ± SD  (min – max) | *p^+^* | **Delta VAS**  Mean ± SD  (min – max) |
| --- | --- | --- | --- | --- |
| **Overall (N=155)** | |  |  |  |
| Male  (n=74) | 24.5 ± 21.9  (0 – 90) | 23.0 ± 21.5  (0 -78) | .174 | 1.6 ± 21.7  (-68 – 63) |
| Female  (n=81) | 34.6 ± 23.6  (0 – 94) | 27.6 ± 21.5  (0 – 78) | **.005** | 7.4 ± 23.2  (-55 – 65) |
| *p** | **.005** | .090 |  | .074 |
| **DPOX (n=52)** |  |  |  |  |
| Male  (n=22) | 21.6 ± 19.5  (0 – 67) | 16.8 ± 12.8  (0 – 50) | **.050** | 4.8 ± 16.8  (-50 – 37) |
| Female  (n=30) | 38.9 ± 23.6  (0 – 83) | 26.9 ± 19.1  (0 – 60) | **.002** | 12.0 ± 18.4  (-30 – 65) |
| *p** | **.010** | .078 |  | .232 |
| **ARGI (n=52)** |  |  |  |  |
| Male  (n=27) | 24.2 ± 20.6  (0 – 70) | 25.0 ± 22.6  (0 – 78) | .224 | -0.8 ± 23.8  (-69 – 38) |
| Female  (n=25) | 33.9 ± 23.1  (0 – 84) | 27 8 ± 17.4  (0 – 73) | .796 | 7.5 ± 26.5  (-55 – 46) |
| *p** | .068 | .309 |  | .158 |
| **CRTL (n=51)** |  |  |  |  |
| Male  (n=25) | 27.4 ± 25.6  (0 - 90) | 26.3 ± 25.7  (0 – 73) | .685 | 1.5 ± 24.2  (-47 – 63) |
| Female  (n=26) | 30.3 ± 24.1  (2 – 94) | 28.3 ± 21.5  (0 – 57) | .484 | 2.0 ± 24.9  (-48 – 44) |
| *p** | .534 | .755 |  | .821 |

* Wilcoxon signed rank test

^+^ Mann-Whitney U-test
