# Supplementary material for: Does a pretreatment with a dentine hypersensitivity mouth-rinse compensate the pain caused by professional mechanical plaque removal? A single-blind randomized controlled clinical trial
Source: Clin Oral Investig. 2020 Oct 23;25(5):3151–60. doi: 10.1007/s00784-020-03643-4 (PMC8060178; doi:10.1007/s00784-020-03643-4)
Supplement: Supplementary file 3 — (DOCX 11 kb) [file 784_2020_3643_MOESM3_ESM.docx]

**Ingredients of Nanaminze mouth-rinse** (Alverde Naturkosmetik, Karlsruhe, D):

Aqua, Sorbitol, Betaine, Aloe Barbadensis Leaf Juice Powder**, Salvia Officinalis Leaf Extract**, Mentha Piperita Oil**, Mentha Spicata Crispa Herb Oil, Mentha Arvensis Leaf Oil, Dipotassium Glycyrrhizate, Sodium Fluoride, Zinc Gluconate, Glycerin, Menthol, Citric Acid, Limonene***, Citral***, Linalool***

**ingredients from certified organic agriculture

***from natural essential oil
